# Supplementary material for: AffyMAPSDetector: a software tool to characterize Affymetrix GeneChip™ expression arrays with respect to SNPs
Source: BMC Bioinformatics. 2007 Jul 30;8:276. doi: 10.1186/1471-2105-8-276 (PMC1959249; doi:10.1186/1471-2105-8-276)
Supplement: Additional file 13 — AffyMAPSDetector v1 source code. AffyMAPSDetector source code. [file 1471-2105-8-276-S13.zip › AffyMAPSDetector_v1_src/doc/jwf/ErrorMessageBox.html]

ErrorMessageBox


|  |  |  |  |
| --- | --- | --- | --- |
| **Overview** \| **Package** \| **Class** \| **Use** \| **Tree** \| **Deprecated** \| **Index** \| **Help** | | |  |
| PREV CLASS   **NEXT CLASS** | **FRAMES**    **NO FRAMES**     **All Classes** |  |  |
| SUMMARY: NESTED \| FIELD \| CONSTR \| METHOD | DETAIL: FIELD \| CONSTR \| METHOD |  |  |


---


## jwf Class ErrorMessageBox

```
java.lang.Object
  java.awt.Component
      java.awt.Container
          java.awt.Window
              java.awt.Dialog
                  javax.swing.JDialog
                      jwf.ErrorMessageBox
```

**All Implemented Interfaces:**: javax.accessibility.Accessible, java.awt.event.ActionListener, java.util.EventListener, java.awt.image.ImageObserver, java.awt.MenuContainer, javax.swing.RootPaneContainer, java.io.Serializable, javax.swing.WindowConstants

---

public class **ErrorMessageBox** extends javax.swing.JDialog implements java.awt.event.ActionListener

Displays a list of error messages and blocks until ok is pressed.

**Author:**
:   Christopher Brind

**See Also:**: Serialized Form

---

**Nested Class Summary**

**Nested classes inherited from class javax.swing.JDialog**

`javax.swing.JDialog.AccessibleJDialog`

**Nested classes inherited from class java.awt.Dialog**

`java.awt.Dialog.AccessibleAWTDialog`

**Nested classes inherited from class java.awt.Window**

`java.awt.Window.AccessibleAWTWindow`

**Nested classes inherited from class java.awt.Container**

`java.awt.Container.AccessibleAWTContainer`

**Nested classes inherited from class java.awt.Component**

`java.awt.Component.AccessibleAWTComponent, java.awt.Component.BltBufferStrategy, java.awt.Component.FlipBufferStrategy`


**Field Summary**

**Fields inherited from class javax.swing.JDialog**

`accessibleContext, rootPane, rootPaneCheckingEnabled`

**Fields inherited from class java.awt.Component**

`BOTTOM_ALIGNMENT, CENTER_ALIGNMENT, LEFT_ALIGNMENT, RIGHT_ALIGNMENT, TOP_ALIGNMENT`

**Fields inherited from interface javax.swing.WindowConstants**

`DISPOSE_ON_CLOSE, DO_NOTHING_ON_CLOSE, EXIT_ON_CLOSE, HIDE_ON_CLOSE`

**Fields inherited from interface java.awt.image.ImageObserver**

`ABORT, ALLBITS, ERROR, FRAMEBITS, HEIGHT, PROPERTIES, SOMEBITS, WIDTH`


**Constructor Summary**

`ErrorMessageBox()`
  
          Construct a dialog with no parent.

`ErrorMessageBox(java.awt.Dialog dialog)`
  
          Construct a dialog with a dialog parent.

`ErrorMessageBox(java.awt.Frame frame)`
  
          Construct a dialog with a frame parent.


|  |  |
| --- | --- |
| **Method Summary** | |
| `void` | `actionPerformed(java.awt.event.ActionEvent ae)`             Handles the ok press. |
| `void` | `showErrorMessages(java.util.List list)`             Show a list of messages and block until ok is pressed. |

**Methods inherited from class javax.swing.JDialog**

`addImpl, createRootPane, dialogInit, getAccessibleContext, getContentPane, getDefaultCloseOperation, getGlassPane, getJMenuBar, getLayeredPane, getRootPane, isDefaultLookAndFeelDecorated, isRootPaneCheckingEnabled, paramString, processWindowEvent, remove, setContentPane, setDefaultCloseOperation, setDefaultLookAndFeelDecorated, setGlassPane, setJMenuBar, setLayeredPane, setLayout, setRootPane, setRootPaneCheckingEnabled, update`

**Methods inherited from class java.awt.Dialog**

`addNotify, dispose, getTitle, hide, isModal, isResizable, isUndecorated, setModal, setResizable, setTitle, setUndecorated, show`

**Methods inherited from class java.awt.Window**

`addPropertyChangeListener, addPropertyChangeListener, addWindowFocusListener, addWindowListener, addWindowStateListener, applyResourceBundle, applyResourceBundle, createBufferStrategy, createBufferStrategy, finalize, getBufferStrategy, getFocusableWindowState, getFocusCycleRootAncestor, getFocusOwner, getFocusTraversalKeys, getGraphicsConfiguration, getInputContext, getListeners, getLocale, getMostRecentFocusOwner, getOwnedWindows, getOwner, getToolkit, getWarningString, getWindowFocusListeners, getWindowListeners, getWindowStateListeners, isActive, isFocusableWindow, isFocusCycleRoot, isFocused, isShowing, pack, postEvent, processEvent, processWindowFocusEvent, processWindowStateEvent, removeWindowFocusListener, removeWindowListener, removeWindowStateListener, setCursor, setFocusableWindowState, setFocusCycleRoot, setLocationRelativeTo, toBack, toFront`

**Methods inherited from class java.awt.Container**

`add, add, add, add, add, addContainerListener, applyComponentOrientation, areFocusTraversalKeysSet, countComponents, deliverEvent, doLayout, findComponentAt, findComponentAt, getAlignmentX, getAlignmentY, getComponent, getComponentAt, getComponentAt, getComponentCount, getComponents, getContainerListeners, getFocusTraversalPolicy, getInsets, getLayout, getMaximumSize, getMinimumSize, getPreferredSize, insets, invalidate, isAncestorOf, isFocusCycleRoot, isFocusTraversalPolicySet, layout, list, list, locate, minimumSize, paint, paintComponents, preferredSize, print, printComponents, processContainerEvent, remove, removeAll, removeContainerListener, removeNotify, setFocusTraversalKeys, setFocusTraversalPolicy, setFont, transferFocusBackward, transferFocusDownCycle, validate, validateTree`

**Methods inherited from class java.awt.Component**

`action, add, addComponentListener, addFocusListener, addHierarchyBoundsListener, addHierarchyListener, addInputMethodListener, addKeyListener, addMouseListener, addMouseMotionListener, addMouseWheelListener, bounds, checkImage, checkImage, coalesceEvents, contains, contains, createImage, createImage, createVolatileImage, createVolatileImage, disable, disableEvents, dispatchEvent, enable, enable, enableEvents, enableInputMethods, firePropertyChange, firePropertyChange, firePropertyChange, getBackground, getBounds, getBounds, getColorModel, getComponentListeners, getComponentOrientation, getCursor, getDropTarget, getFocusListeners, getFocusTraversalKeysEnabled, getFont, getFontMetrics, getForeground, getGraphics, getHeight, getHierarchyBoundsListeners, getHierarchyListeners, getIgnoreRepaint, getInputMethodListeners, getInputMethodRequests, getKeyListeners, getLocation, getLocation, getLocationOnScreen, getMouseListeners, getMouseMotionListeners, getMouseWheelListeners, getName, getParent, getPeer, getPropertyChangeListeners, getPropertyChangeListeners, getSize, getSize, getTreeLock, getWidth, getX, getY, gotFocus, handleEvent, hasFocus, imageUpdate, inside, isBackgroundSet, isCursorSet, isDisplayable, isDoubleBuffered, isEnabled, isFocusable, isFocusOwner, isFocusTraversable, isFontSet, isForegroundSet, isLightweight, isOpaque, isValid, isVisible, keyDown, keyUp, list, list, list, location, lostFocus, mouseDown, mouseDrag, mouseEnter, mouseExit, mouseMove, mouseUp, move, nextFocus, paintAll, prepareImage, prepareImage, printAll, processComponentEvent, processFocusEvent, processHierarchyBoundsEvent, processHierarchyEvent, processInputMethodEvent, processKeyEvent, processMouseEvent, processMouseMotionEvent, processMouseWheelEvent, remove, removeComponentListener, removeFocusListener, removeHierarchyBoundsListener, removeHierarchyListener, removeInputMethodListener, removeKeyListener, removeMouseListener, removeMouseMotionListener, removeMouseWheelListener, removePropertyChangeListener, removePropertyChangeListener, repaint, repaint, repaint, repaint, requestFocus, requestFocus, requestFocusInWindow, requestFocusInWindow, reshape, resize, resize, setBackground, setBounds, setBounds, setComponentOrientation, setDropTarget, setEnabled, setFocusable, setFocusTraversalKeysEnabled, setForeground, setIgnoreRepaint, setLocale, setLocation, setLocation, setName, setSize, setSize, setVisible, show, size, toString, transferFocus, transferFocusUpCycle`

**Methods inherited from class java.lang.Object**

`clone, equals, getClass, hashCode, notify, notifyAll, wait, wait, wait`

**Constructor Detail**

### ErrorMessageBox

```
public ErrorMessageBox()
```

:   Construct a dialog with no parent.

---


### ErrorMessageBox

```
public ErrorMessageBox(java.awt.Frame frame)
```

:   Construct a dialog with a frame parent.

---


### ErrorMessageBox

```
public ErrorMessageBox(java.awt.Dialog dialog)
```

:   Construct a dialog with a dialog parent.


**Method Detail**

### actionPerformed

```
public void actionPerformed(java.awt.event.ActionEvent ae)
```

:   Handles the ok press.

    :   **Specified by:**: `actionPerformed` in interface `java.awt.event.ActionListener`

---


### showErrorMessages

```
public void showErrorMessages(java.util.List list)
```

:   Show a list of messages and block until ok is pressed.

    :   **Parameters:**: `list` - a List of String objects.


---


|  |  |  |  |
| --- | --- | --- | --- |
| **Overview** \| **Package** \| **Class** \| **Use** \| **Tree** \| **Deprecated** \| **Index** \| **Help** | | |  |
| PREV CLASS   **NEXT CLASS** | **FRAMES**    **NO FRAMES**     **All Classes** |  |  |
| SUMMARY: NESTED \| FIELD \| CONSTR \| METHOD | DETAIL: FIELD \| CONSTR \| METHOD |  |  |


---
